# Effects of Type 1 Diabetes Mellitus on Linear Growth: A Comprehensive Review

**DOI:** 10.7759/cureus.45428

**Published:** 2023-09-17

**Authors:** Indrayani Jadhav, Swarupa Chakole

**Affiliations:** 1 Medicine and Surgery, Jawaharlal Nehru Medical College, Datta Meghe Institute of Higher Education and Research, Wardha, IND; 2 Community Medicine, Jawaharlal Nehru Medical College, Datta Meghe Institute of Higher Education and Research, Wardha, IND

**Keywords:** mauriac syndrome, insulin therapy, linear growth, insulin-like growth factor, growth hormone, type 1 diabetes mellitus

## Abstract

Type 1 diabetes mellitus (T1DM) has a significant effect on the growth of children. The disease has a negative effect on growth when considered in relation to the time period and metabolic control. Studies in this review have suggested debilitated growth in children with T1DM and have a few anomalies in the growth hormone (GH)-insulin-like growth factor-1 (IGF-1) axis when compared to fit children. Some studies show that children with T1DM were taller before the onset of the disease and during early diagnosis. Moreover, the linear growth depends on the interaction between the gonadotropin hormone, luteinizing hormone (LH), follicle-stimulating hormone (FSH), and sex steroid hormones axis and GH-IGF-1; there’s a rise in GH during puberty, which has an effect on the estrogen and testosterone, which leads to the pulsatile secretion of GH, this increment leads to insulin resistance. These studies suggest short stature in girls, and some suggest in both. The final height in boys was unchanged, but a slight decline was observed in girls. This review aims to provide the latest understanding of impaired height in children with T1DM. The most accepted and effective treatment of impaired growth is the administration of long-acting insulin or continuous rapid-acting insulin. However, height was affected by the administration of good basal insulin at puberty and was unaffected by the continuous subcutaneous insulin injection. Hence, new technologies are the therapeutic regimen in children, especially the prepubertal age group; it will be interesting to see their effects on growth patterns in these children with T1DM.

## Introduction and background

Type 1 diabetes mellitus (T1DM) is a chronic debility with short-term as well as long-term effects. Type 1 diabetes has been rising worldwide at 3-5% annually. T1DM majorly occurs due to autoimmunity against the beta cells, which produces insulin and destroys beta cells, leading to complete or near-total insulin deficiency. Other causes include genetics, exposure to viruses, or other environmental factors. T1DM commonly occurs before age 30; autoimmunity against the beta cell can be developed anytime throughout a lifetime. It has been known that the immune system significantly influences the genesis of illness; the antibodies are detected, which identify the beta-cell component of the pancreas and the individual’s blood insulin levels a year before the onset of T1DM. Endocrinological, dietary, and psychological variables influence the complicated physiological process of a child’s growth. The effects of T1DM are still unclear. Some studies show that growth is impaired in chronic hyperglycemia [1]. Early onset and long duration of diabetes mellitus in children are linked to poor growth. Mauriac syndrome is one such example. Mauriac syndrome is now a rare condition of poorly controlled T1DM, usually characterized by hepatomegaly and growth failure. The pathogenesis of growth failure in this syndrome is unclear but is said to be multifactorial, hyperglycemia being one such factor. This syndrome is rare, and the growth prognosis has improved due to systemic glycemic control and treatment regimens. T1DM pediatric patients usually present with some deformity in the growth hormone (GH) or insulin-like growth factor (IGF) axis in comparison with the fit pediatric age group population [2]. This article's primary objective is to summarize the literature on how T1DM affects children's and adolescents' growth.

## Review

Methodology

**Figure 1:**
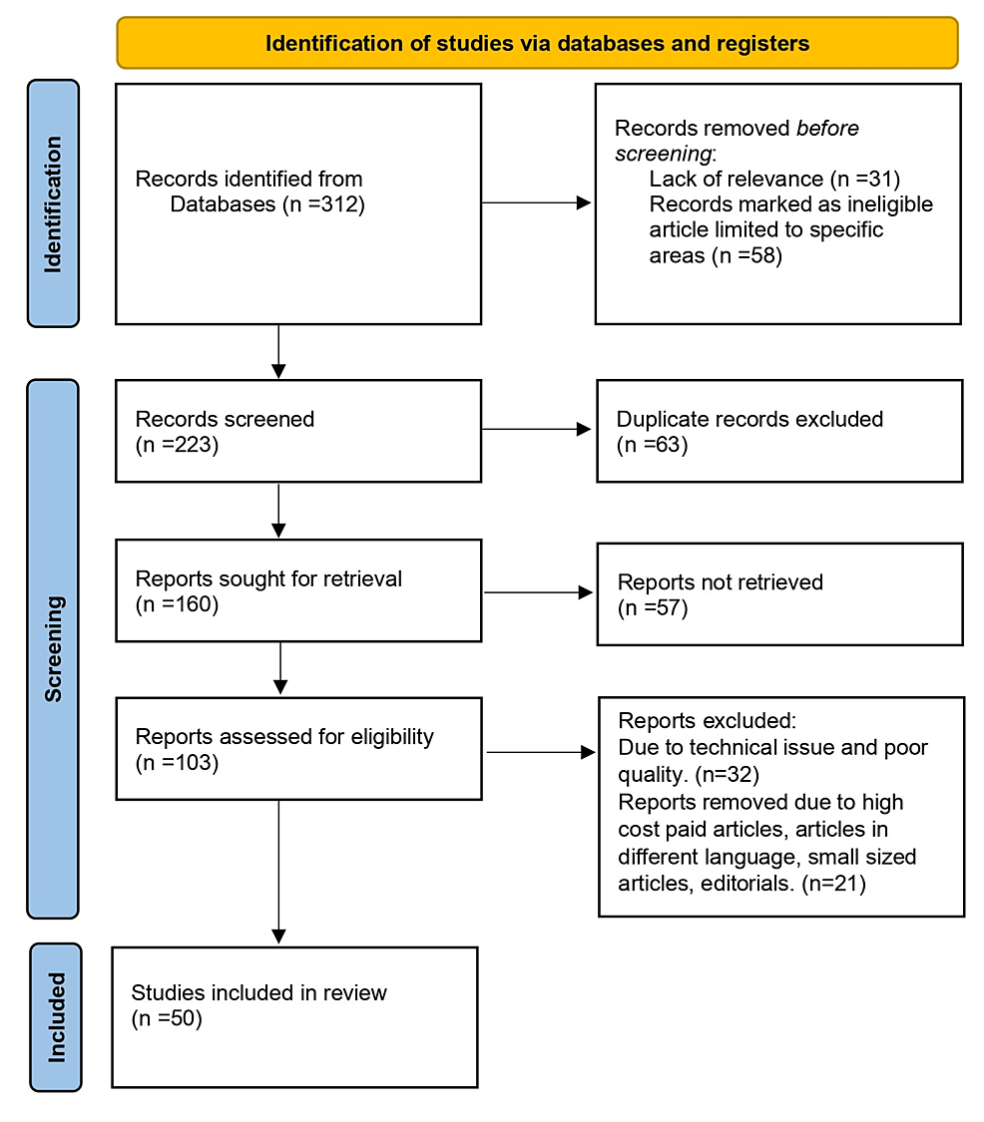
PRISMA methodology PRISMA, Preferred Reporting Items for Systematic Reviews and Meta-Analysis

Using the electronic databases PubMed, MEDLINE, Embase, Google Scholar, and ResearchGate, a search of English-language literature was done. It was also the subject of a different search. The query term was “type one diabetes mellitus” OR “insulin dependent diabetes mellitus” OR “diabetes mellitus”; “linear growth” OR “linear height”; “insulin therapy” OR “treatment modalities.” The inclusion criteria involve articles with studies conducted exclusively on T1DM, autoimmune T1DM, the effects of it on linear growth, and insulin therapy; studies conducted preceding 10 years are also included. The exclusion criteria involve articles with the other effects of T1DM, other causative factors of T1DM, and adults having T1DM. The articles are excluded due to lack of relevance, records marked as ineligible articles limited to specific areas, technical issues and poor quality, high-paid articles, and small-sized articles. Figure 1 highlights the PRISMA method's use in research methodology.

Impaired growth in T1DM

Growth plate chondrocytes are orderly differentiated throughout the longitudinal growth of bones from the inactive to the rapid division to the hypertrophic stage. Vascular invasion and osteoblastic invasion occur eventually, causing bone elongation and the formation of new bone [3]. This process depends on the GH-insulin-like growth factor1 (IGF-1) axis. GH directly enhances the proliferation of chondrocytes and is one of the most essential regulating components for the development of bones in children and adolescents [4]. GH has a fluctuating production with age, characterized by less secretion before puberty, a spike throughout puberty, and decreases with advancing years [5]. IGF-1 and insulin-like growth factor-2 (IGF-2) peptides constitute most of the actions of GH. IGF-1 has an association with particular insulin-like growth factor binding proteins (IGFBP) and is reversible. IGFBP have been divided into six different types so far [6]. Out of these six IGFBPs, the third (IGFBP-3) is the primary circulating binding protein, which binds with IGF-1 in a trinary compound with an acid-labile component and inhibits interplay between IGF-1 and its receptors, where GH enhances its production [7]. Insulin has various effects on the GH/IGF-1 axis, including altering the GH receptor's (GHR) expression in the liver and interaction with GH post-receptor signaling to control the production of IGF-1 and IGFBPs in the liver [8,9]. It has been shown that T1DM patients exhibit specific modifications in the GH/IGF-1 axis, particularly during puberty. These modifications include excess secretion of GH, a reduction in IGF-1 and IGFBP-3 levels in the blood, and a rise in IGFPB-1 levels [8,10-16]. Reduced intraportal insulin concentration is likely the cause of reduced IGF-1 serum levels. A child and an adolescent with T1DM and inadequate metabolic control have been found to have a number of abnormalities [17]. Furthermore, it is interesting to note that in T1DM patients, there is decreased negative feedback to the pituitary due to low IGF-1 levels, which leads to increased secretion of GH [18]. The primary contributing cause to the insulin resistance that characterizes T1DM before puberty is an increased level of GH concentration. Additionally, a low intraportal insulin concentration causes an increase in the synthesis of IGFBP-1, which inhibits the bioactivity of IGF-1 [14,19,20]. Insulin manages the availability of GHRs by alteration and downstream signaling to regulate the hepatic GH responses [9]. Changes in IGF1 and IGFBP concentrations are, therefore, said to be associated with reduced insulin levels in the portal vein, as the therapeutic supply of insulin in diabetes comes through the subcutaneous route. These abnormalities improve with insulin administration or therapy intensification but do not return to normal [21,22].

Growth before puberty and at the onset of T1DM

After the insulin was discovered, it was observed that the children were taller at the time of T1DM onset than the unaffected children [23]. Some observed that this effect was more significant in boys or children between the ages of five to 10 years who have developed T1DM but not in those with the early or late start of T1DM [23-25]. In a cohort, Bonfig and his colleagues confirmed this phenomenon by observing children in Germany and Austria who were diagnosed with T1DM; the average height standard deviation score (SDS) was 0.22±1.00 at the beginning of the ailment, which is greater than the standard country benchmark. The results look more prominent in kids diagnosed at a young age, with average height SDS values of 0.30±1.00, 0.26±0.99, and 0.09±0.98 among kids with the ailment from age zero to five years, six to 11 years, and 12 to 17 years in the written order [26]. According to research by the European Diabetes Centers Study of Complications In Patients With Insulin-Dependent Diabetes Mellitus (EURODIAB), compared to healthy children, patients diagnosed before 15 had greater SDS, higher weight at one month old, and higher body mass index (BMI) at six months [27]. According to three upcoming studies of high-risk patients for T1DM based on their HLA genotype or ancestral history, accelerated growth is connected to islet autoimmunity. In cohorts Babies Development and Diabetes (BABYDIAB) and BABYDIET, infantile BMI was inversely corresponding to islet autoimmunity with a hazard ratio (HR) of 0.60 per two standard deviations (SD) increase in age [28]. The development of T1DM (HR 3.34) as well as the initiation of islet autoimmunity were both positively linked with height velocity in the Diabetes Autoimmunity Study in the Young (DAISY) cohort, which measures the anthropometric parameters taken into account after the child turned two [29]. In the Environmental Determinants of Diabetes in the Young (TEDDY) cohort of high-risk patients, immunity against one's own body was not correlated with height SDS at one year [30]. To explain this increase in height at T1DM onset, it is suggested that there is augmented IGFBP-3 proteolysis and subsequently reduced secretion of insulin during the prediabetic period, which results in increases in the availability of IGF-1 [31]. The growth pattern was influenced by metabolic control and suggested that height velocity after the diagnosis of T1DM directly correlated with the residual activity of pancreatic beta cells, which was evaluated by estimating the C-peptide levels [31]. Another study suggested a depletion in growth velocity and height SDS in children with T1DM after the onset before puberty starts [31,32]. Abnormalities in the hypothalamic-pituitary-IGF-1 axis are closely related to variations in height SDS in prepubertal age; thus the hypothalamic-pituitary-IGF-1 axis is affected by T1DM [33]. 

Growth during puberty

Puberty is a crucial phase in the growth of an individual that results in the attainment of the ultimate height of a grown person and the acquisition of reproducing potential. The pubertal growth spurt, which occurs at this stage of puberty, is controlled by the complex interaction of GH/IGF-1 and the axis of gonadotropin-releasing hormone (GnRH), luteinizing hormone (LH), follicle-stimulating hormone (FSH), and sex steroid hormones. With an expected rise in GH and IGF-1 levels throughout the pubertal age, both estrogens and testosterone significantly impact linear growth and enhance the intensity of fluctuating secretions of GH [34,35]. This increment of pulsatile GH secretion and overnight GH concentration is much higher in T1DM patients at puberty than in the control adolescents [18,36]. The rise in the level of GH in serum is considered the most important cause of high insulin requirement and insulin resistance in T1DM patients, being resistant at all stages of puberty [37]. A study that observed the growth in patients with T1DM at puberty showed an impaired pubertal spurt only in girls with ultimate short height less than the parental target height [38]. Another study suggested that the impaired pubertal spurt was only seen during pubertal age, in males only [39]. Plamper et al. studied patients with T1DM aged seven to 16 years. They observed a decline in mean maximal growth velocity in males, while females, after attaining the highest height velocity, showed a comparatively faster decline in pubertal growth spurt [40]. T1DM patients with onset before five years of age had significant height loss during puberty and had a relation between comparatively poor growth and early age of onset [41]. The serum IGF-1 levels were lower in adolescents with T1DM (both boys and girls) at puberty, but there was no relation between the IGF-1 levels and GH binding protein [42].

Final height

Even though the height was lost at the onset of T1DM, an impaired height was never observed. Another study that was conducted suggested that children with diabetes had impaired ultimate height during the period before intense insulin therapy [5]. Boys with diabetes had standard ultimate heights, whereas females with diabetes had a slight decline in final height [38]. The final height SDS of the 80 Oxford research participants was -0.06±1.18, which does not differ remarkably from the mid-parental height SDS of ±0.12±1.00. The early onset of T1DM in children was associated with slightly reduced height [24]. This data was confirmed, showing linear growth reduction being distinct in patients diagnosed before puberty, with prepubertal stage diabetes onset being lower than the rest of the stages. This study also showed that in children with onset of diabetes before puberty with poorly controlled T1DM, a noticeable decline was seen in height compared to children with reasonable control. Children with T1DM were observed to be taller at the time of diagnosis when they were between the ages of five and ten, and later, loss of height canceled out the prior increase in height [43].

Mauriac syndrome

Mauriac syndrome is an extreme growth failure in T1DM. It is usually present in addition to growth failure; delayed puberty; hepatomegaly; dwarfism; dyslipidemia; reduction in IGF-1; and Cushinoid features consisting of a round face, posterior neck hump, and spontaneous gain of weight. Mauriac syndrome has been associated with insufficient nutrition, fragile glycemic control, and poor insulin compliance in children and adolescents. There has been a reduction in the frequency of blood glucose monitoring and blood glucose control and glycated hemoglobin or hemoglobin A1c (HbA1c) [44]. This syndrome has a higher risk for adolescents but can occur at any age if proper metabolic control is not obtained. T1DM patients obtain a poorly controlled state of hyperglycemic periods with sometimes hyperinsulinemia, which causes glycogen storage in hepatocytes. Deficient salinization because of improper glycemic control results in lipolysis and ketosis. Ketosis led to the synthesis of cortisol, which influenced the release of fatty acids and hyperglycemia. Muariac syndrome was comparatively more common in adolescent girls. With proper insulin therapy, these features are reversed, with intensive insulin therapy. Due to multiple factors, the pathophysiology of growth retardation in Muriac syndrome is unclear. Due to insufficient glucose in the tissues in T1DM, lowered levels of IGF-1 and GH and increased cortisol levels might have delayed growth and puberty. The growth failure and delayed puberty in Mauriac syndrome can be improved with glycemic control [45].

The effect of insulin therapy on the linear growth of patients with T1DM

One of the treatment goals of effective T1DM therapy is to ensure normal development during childhood and adolescence. Administering long-lasting insulin or continuous rapid-acting insulin infusion, administered subcutaneously to meet basal needs, is the standard of treatment for diabetic patients. Recently, a new regimen has come into being, consisting of multi-dose injection (MDI) or, as mentioned earlier, continuous subcutaneous insulin infusion (CSII), with routine monitoring of blood sugar, has resulted in reasonable metabolic control in T1DM patients, but the effect of this therapy on height is still unclear [46]. Another study demonstrated that height SDS is affected by administering good basal insulin at puberty while using CSII does not affect height [34,47]. In one group, treatment was started in 12 patients of T1DM with glargine insulin treatment, which resulted in decreased levels of IGFBP-3 nocturnally, with increased levels of IGF-1 and improved levels of HbA1c compared to insulin protamine Hagedorn insulin. At the same time, on the counter side, it was seen that there was no effect of glargine insulin on IGF-1, but height gain was observed in children treated with detemir insulin [48,49]. In a study conducted, thirty T1DM male patients (both children and adolescents) were compared with thirty healthy children; these patients were split into three batches: group 1 consisting of prepubertal, group 2 consisting of children who have attained puberty, and group 3 consisting of post-pubertal children. Mean HbA1c readings in prepubertal and pubertal children aligned with the optimal metabolic control possible after receiving intensive treatment. In pubertal and post-pubertal children, HbA1C was comparatively greater than the healthy children but was lesser than the exclusive control value. There was no variation in serum IGFBP-3 and IGF-1 levels between these three groups and healthy controls, suggesting a normal GH-IGF-1 axis in T1DM patients with reasonable control in metabolism. Height had no significant change in the three groups compared to the healthy children, referring to average growth in patients with intensive insulin therapy. This study suggests that planned and cautious insulin administration from the onset and diagnosis of the disease can cut short the anomalies in physiological growth in children and adolescents with T1DM [50]. The CSII controls HbA1c levels and enhances linear growth but simultaneously has a few limitations as it leads to chronic peripheral hyperinsulinization, which is the main factor of rise in BMI, usually before pubertal years. As increased levels of portal insulin are required for the maintenance of normal serum growth factors levels and growth promotion, in T1DM patients, subcutaneous insulin does not restore the normal intraportal levels of insulin [47].

Table 1 shows height SDS, IGF-I, IGFBP-3, BMI, HbA1c, cholesterol, and triglycerides in prepubertal (group 1), pubertal (group 2), and post-pubertal (group 3) children with T1DM and in age-matched controls.

**Table 1:** Height SDS, IGF-1, IGFBP-3, BMI, HbA1c, cholesterol, and triglycerides in prepubertal (group 1), pubertal (group 2), and post-pubertal (group 3) children with T1DM and in age-matched controls [50]. SDS, standard deviation score; IGF-1, insulin-like growth factor 1; IGFBP-3, insulin-like growth factor binding protein 3; HbA1c, hemoglobin A1c; BMI, body mass index

	Group 1			Group 2			Group 3		
Parameters	Type1 Diabetes Mellitus	Control	P	Type1 Diabetes Mellitus	Control	P	Type1 Diabetes Mellitus	Control	P
Height SDS	0.68±0.9	0.46±0.9	0.76	0.15±1.6	0.17±1.33	0.12	0.19±0.0010.8	0.18±0.7	0.63
IGF-1	180.3±120.14	176.6±42.63	0.82	379.8±146.1	471±166	0.27	364±144	359±53.1	0.9
IGFBP-3	3022±902.2	3173±623	0.46	3376.3±427.4	4116.2±703.5	0.34	3364.6±605.2	3772.9±254.8	0.52
BMI	19.8±3.3	21.4±8.1	0.89	21.7±2.4	20.8±4.1	0.46	23.1±9.2	24.1±6.7	0.48
HbA1c (%)	7±0.9	4.9±0.2	<0.001	7.1±1.2	2.0±0.2	<0.001	6.8±0.8	5.0±0.2	<0.001

## Conclusions

The most prevalent form of chronic metabolic illness in children is T1DM. The impaired growth in children and adolescents with the disease is influenced by various factors. As mentioned in the review, the GH-IGF-1 axis plays a vital role in the growth of the bones; alteration in the axis was observed in children with T1DM. Prior to the onset of puberty and at the onset of the disease, children were initially taller when diagnosed early. However, this initial increase in height was usually followed by a decrease in the velocity of growth after the diagnosis; this growth pattern was influenced by metabolic control. During puberty, with an increment in GH and IGF-1 levels, the sex hormones have a significant impact on the linear growth and enhance the intensity of fluctuation in GH; this rise in GH is higher in T1DM children and is considered the cause of high insulin requirement and insulin resistance. The final height of an adult having T1DM was observed to be decreased when compared to children with good metabolic control. Effective insulin therapy is of the utmost importance to ensure normal development during childhood and adolescence. It can be done by either long-lasting insulin or continuous rapid-acting insulin infusion. A new regimen, MDI or CSII, has given reasonable metabolic control in T1DM children, but the effect of these therapies on height is still unclear. Proper management, especially at the time of diagnosis and onset of disease, can help prevent growth abnormalities. The impaired growth in T1DM is a complex, multifactorial problem influenced by the age of diagnosis of the disease, the metabolic control, and the effective therapy. It is important to assess these factors and carry out effective management strategies to promote normal growth and development in children with T1DM.
